# Quantitative comparison and evaluation between aerial and underground parts of *Gentiana straminea* through simultaneous determination of five major compounds by RP-HPLC

**DOI:** 10.1016/j.heliyon.2024.e29232

**Published:** 2024-04-15

**Authors:** Junlei Hao, Jiang Zhou, Pengcheng Lin, Jiang Wu

**Affiliations:** Key Laboratory for Tibet Plateau Phytochemistry of Qinghai Province, College of Pharmacy, Qinghai Nationalities University, Xining, 810007, China

**Keywords:** Secoiridoids, Over-collection, Tibetan Plateau, Principal component analysis

## Abstract

Herbal *Gentiana straminea* Maxim. (Family Gentianaceae), “Ma Hua Jiao” in Chinese, is a commonly used Chinese medicine. Secoiridoids and flavonoids have been identified as the major active components of herbal medicines used in the treatment of hepatitis, rheumatism and many other diseases. It is the overharvesting of the roots of this plant for medicinal purposes that has led to a drastic decline in its population. In the present study, the above and below ground parts of Gentian Bitter Glycine were quantitatively compared and evaluated for the determination of the major active constituents. Five major compounds, loganic acid, swertiamarin, gentiopicroside, sweorside and isoorientin, were extracted by solvent extraction technique and analyzed by Reversed-phase High Performance Liquid Chromatography (RP-HPLC). By analysing the principal components and calculating the composite scores, the results show that the aboveground component in different areas ranked higher compared to the underground component, with the former being able to substitute to some extent for the latter's underground component. Finally, based on hierarchical cluster analysis, we identified the ideal natural growing region for aerial parts of *G. straminea* distributed on the Qinghai-Tibetan Plateau. The significance of this work is that we can balance the demand for herbs with environmental preservation by selectively picking the aerial parts, which can regrow next year, instead of removing the whole plant. It protects the fragile ecological environment of the Tibetan Plateau and is important for sustainable development.

## 1 Introduction

*Gentiana straminea* Maxim. [1,2], a very famous Chinese folk herb of the family Gentianaceae, commonly known as “Ma Hua Jiao,” is quite frequently used to treat diverse diseases, such as fungal and bacterial infections, jaundice, hepatitis, constipation, pain and rheumatism [3,4]. This medicine contains several bitter compounds, mainly secoiridoids and flavonoids, which are regarded as the major active components [[5], [6], [7], [8]]. Flavonoids have been reported to have antioxidant activity and antimicrobial potential [[9], [10], [11]], while its ethanol extract produces an inhibitory effect on anthrax bacillus, staphylococci, typhoid bacillus, pneumococci, bacillus dysenteriae, and vibrio [12,13]. Historically, Chinese physicians prepared this medicine using the root of *G. straminea*, while Tibetan doctors typically used the entire plant. Following our previous phytochemical investigation of *G. straminea* [[14], [15], [16], [17], [18]], we determined that its major phytochemical constituents are loganic acid, swertiamarin, gentiopicroside, sweorside, and isoorientin. These active constituents, especially gentiopicroside, a major bitter secoiridoid glycoside widespread in the Gentianaceae family, were found to be capable of protecting against hepatitis by inhibiting the production of tumor necrosis factor [19,20], and by promoting the secretion of gastric juice [21]. Another important biologically active compound is swertiamarin, which was observed to improve skin function, promote hair growth, and function as an antipsychotic [21,22]. Loganic acid was observed to exhibit analgesic, anti-inflammatory, and febrifuge pharmacological effects [23,24]. Both sweorside and isoorientin exhibit the ability to suppress chemically and immunologically induced hepatic injuries [[25], [26], [27]].

This species is a perennial herb distributed in the high mountains and alpine environment of the Qinghai-Tibet Plateau, at altitudes from 2, 000 to 5, 000 m [28]. The alpine plants growing in these regions are often subject to serious stresses to which they have had to adapt, including low temperatures and strong solar radiation [29]. In recent years, the natural resources of *G. straminea* have declined dramatically due to overharvesting of the flowering plants, without leaving sufficient seeds to maintain its population. This species has now been listed as endangered by the local governments, and further harvesting has been prohibited in some areas. In order to devise adequate conservation and management strategies for this species, it is important to seek new technology that will allow us to balance the continued utilization of this resource with the need to protect the fragile ecological environment of Qinghai-Tibet Plateau.

The medicinal importance and lack of resources of the plant prompted our interest in finding alternatives to one-time destructive harvesting; specifically, the selective harvesting of aerial parts of *G. straminea* while retaining its underground parts for continued perennial growth and maximum resource utilization. A number of published methods have focused on the qualitative and quantitative determination of cyclic enol ether terpenoids and flavonoids in *G. straminea* using Liquid Chromatography (LC) or Liquid Chromatography-Mass Spectrometry (LC-MS) [30,31]. However, few of these methods aim at quantitatively comparing and evaluating the above-ground and below-ground parts of the plant by determining the major components. The purpose of this paper was to statistically compare the concentrations of the five major active constituents in the aerial and underground parts of *G. straminea* using materials collected from fifteen different high-altitude populations on the Qinghai-Tibetan Plateau. Principal component analysis in SPSS software has been previously used as a method to analyze and explain data prior to HPLC analysis. Results of this investigation indicated that the aerial parts yielded considerable concentrations of the five active principles that are also found in the underground parts, which are currently harvested for the treatment of diverse ailments. Furthermore, hierarchical clustering analysis [32] also helped to identify the most suitable regions for producing the aerial parts of *G. straminea*.

## 2 Methods

### 2.1 Chemicals, reagents and materials

**Fig. 1:**
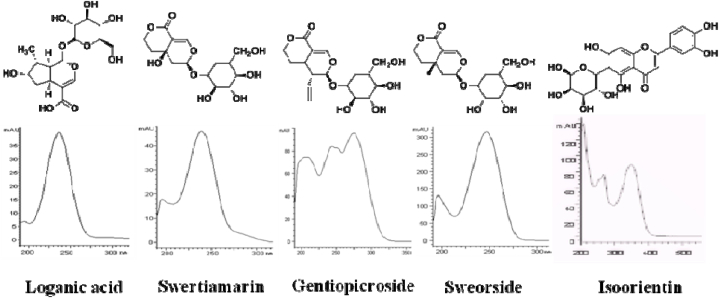
Structures of investigated five compounds and its UV chromatograms.

Some findings have shown that polar organic solvents are the most efficient in the extraction of flavonoid and phenolic components [33]. In our laboratory, methanol was used for the extraction of the main components and the isolation and purification of the marker components, loganic acid, swertiamarin, gentiopicroside, sweorside, and isoorientin, were carried out. The purity of each compound was determined by HPLC to be greater than 98 %. Compound structures were confirmed by comparing their UV, MS, ^1^HNMR and ^13^CNMR data with data from the literature [[34], [35], [36], [37], [38]]. Loganic acid was generously provided by the Northwest Institute for Plateau Biology, Chinese Academy of Sciences (Xining, China). The chemical structures of these reference compounds are summarized in Fig. 1.

HPLC-grade methanol was purchased from Yuwang Co., Ltd (Shandong, China). The other chemicals and reagents used in the experiment were of Chromatographic or analytical grade quality.

Natural *G. straminea* was obtained from different counties of Qinghai province of China. Cultured *G. straminea* was obtained from Huangnan prefecture of Qinghai province of China (Table 1). The identities of these *G. straminea* samples were confirmed by a member of our research group, Professor Pengcheng Lin. All corresponding voucher specimens were deposited at the experimental center of the college of Chemistry and Life Science, Qinghai University for Nationalities, Xining, China. Dried samples were ground to 0.30–0.45 mm.

**Table 1:** Summary for the tested samples of *G. straminea*.

NO.	Code	Samples	Sourses	Altitude	Collection Date
1	MY	*G. straminea*	Menyuan county, qinghai, China	3100 m	2008.8
2	ZK-1	*G. straminea*	Zeku county1, qinghai, China	3350 m	2008.8
3	HZ	*G. straminea*	Huzhu county, qinghai,China	2200 m	2008.8
4	NQ	*G. straminea*	Nangqian county, qinghai,China	3700 m	2008.8
5	ZK-2	*G. straminea*	Zeku county2, qinghai, China	3350 m	2008.8
6	TD	*G. straminea*	Tongde county, qinghai, China	3400 m	2008.8
7	HN-1	*G. straminea*	Henan county1, qinghai, China	3200 m	2008.8
8	YS-1	*G. straminea*	Yushu county1, qinghai, China	3500 m	2008.8
9	HN-2	*G. straminea*	Henan county2, qinghai, China	3200 m	2008.8
10	QL-1	*G. straminea*	Qilian county1, qinghai, China	3300 m	2008.8
11	TR-1	*G. straminea*	Tongren county1, qinghai, China	3200 m	2008.8
12	HNP^a^	*G. straminea*^a^	HuangnanPrefecture, qinghai, China	3150 m	2008.8
13	QL-2	*G. straminea*	Qilian county2, qinghai, China	3300 m	2008.8
14	TR-2	*G. straminea*	Tongren county2, qinghai, China	3200 m	2008.8
15	YS-2	*G. straminea*	Yushu county2, qinghai, China	3500 m	2008.8

a Samples of *G. straminea* were cultured, while other species were wild.

### 2.2 Sample preparation

The Analytes were first dissolved in methanol solution at approximately 5 mg/mL as a stock solution. Aliquots of the stock solution were transferred to a 2-mL volumetric flask and methanol added to obtain the desired concentration.

An accurately weighed sample (0.50 g) of ground powder (0.30–0.45 mm) was transferred into a 25 mL round-bottomed flask with stopper, and 10 mL methanol was added. The flask was immersed in an ultrasonic water-bath (Kunshan Instrumental, Kunshan, Zhejiang Province, P.R.China) and sonicated for 30 min at 30 °C. The sample was extracted twice and the extracts combined. Finally, the solution was filtered into a 25 mL volumetric flask, followed by addition of methanol to a specified volume, and filtration through a 0.45 μm filter (Agilent Technologies, USA), prior to HPLC analysis.

### 2.3 HPLC analysis

Analysis was performed on an Agilent Series 1100 liquid chromatograph equipped with a vacuum degasser, a quaternary pump, and a diode-array detector (DAD), connected to an Agilent ChemStation running ChemStation software. A Hypersil ODS column (250 mm × 4.6 mm, 5 μm) was used. The standards and samples were separated using a gradient mobile phase consisting of water with 3 mM phosphate (A) and methanol (B). The elution conditions applied were as follows: 0–10 min, isocratic 20 % B; 10–30 min, linear gradient 20–50 % B; and 30–35 min, 50 % B isocratic. The column was then reconditioned with 20 % B isocratic for 15 min after washing for 10 min with 100 % B. The flow rate was 1 mL/min and the injection volume was 10 μL. The column temperature was maintained at 35 °C. The analytes were monitored at 254 nm.

### 2.4 Data analysis

Principal Components Analysis (PCA) is a useful statistical technique and a way to identify patterns in data by expressing the data in such a way as to highlight their similarities and differences. Since patterns in data can be difficult to identify in data of high dimensions, where the luxury of graphical representation is not available, PCA is a powerful alternative tool for data analysis. The other main advantage of PCA is that once you have identified patterns in the data, data can be compressed by reducing the number of dimensions, without significant loss of information. For our analyses, PCA was performed on sets of data using SPSS 15 software for Windows (SPSS Inc., USA).

Hierarchical clustering analysis (HCA) was also performed using SPSS 15 for windows (SPSS Inc., USA). HCA is comprised of a number of “procedures” – graphical, statistical, reporting, processing and tabulating procedures – that enable simple and rapid data evaluation. Between-groups Linkage, a very efficient method for the analysis of variance between clusters, was applied, and the Squared Euclidean distance was selected as a measurement for hierarchical clustering analysis.

## 3 Results

### 3.1 Calibration curves and recovery

For every calibration curve, the calibration concentrations were back-calculated from the peak area of the analytes (Table 2). Deviation from the nominal concentration defined the accuracy (Table 3).

**Table 2:** Linear regression data, LOD and LOQ of investigated compounds from *G. straminea*.

Analytes	Linear regression data			LOD (ng)	LOQ (ng)
	Regressive equation	Linear range(mg/mL)	R^2^		
Loganic acid	y = 173.4x-122	0.364–2.18	0.9999	1.20	3.0
Swertiamarin	y = 195.6x-54.0	0.275–1.65	0.9999	0.58	1.8
Gentiopicroside	y = 1002x+34.4	0.614–3.68	0.9999	0.50	1.3
Sweorside	y = 5744x-195	0.0656–0.394	0.9999	0.84	2.1
Isoorientin	y = 1730x-193	0.0899–0.539	0.9999	0.48	1.5

R^2^= Squares of correlation coefficients for the standard curves.

**Table 3:** Recoveries for the assay of the five compounds in *G. straminea*.

Analytes	Original(mg)	Spiked(mg)	Found(mg)	Recovery (%)	RSD (%)
Loganic acid	0.888	0.910	1.820	102.4	0.50
Swertiamarin	0.627	0.688	1.320	100.7	1.10
Gentiopicroside	1.560	1.535	3.100	100.7	0.55
Sweorside	0.145	0.164	0.310	100.6	0.33
Isoorientin	0.216	0.225	0.440	99.6	0.49

a, Data calculated from the average of five determinations.

Recovery (%) = 100*(amount found-original amount)/amount spiked.

RSD (%) = 100*SD/mean.

A recovery test was used to evaluate the accuracy of the developed method. Known amount of the five analytes were added to approximately 0.50 g of *G. straminea* powder, and then extracted and analyzed as described above. Each sample was analyzed five times. The recovery was calculated based on the total amount of the individual, investigated components, and the average recoveries were determined by the following formula: recovery (%) = first amount found/total amount of individual investigated components × 100 %.

### 3.2 Limit of detection and quantification

The LOD was defined as the concentration of analyte giving S/N = 3, and the LOQ as that giving S/N = 10. The LOD and LOQ values of the investigated compounds are summarized in Table 2.

### 3.3 Precision, repeatability and stability

The variability test was chosen to determine the precision of the developed assay. The intra-day precision was examined on the mixed standards solutions for six replicates within a single day, and the relative standard deviation (RSD) taken as a measure of precision was <1 % of the expected concentration. The repeatability of the developed method for all investigated compounds was determined based on six trials. The repeatability was presented as RSD (*n* = 6). Stability of sample solution was tested, by analysis of the sample solution every 2 h for 12 h. Variation was expressed as RSD (*n* = 3). These results are summarized in Table 4.

**Table 4:** Precision, repeatability and stability.

Analytes	Precision (RSD,%,n = 6)	Repeatability (RSD,%,n = 6)	Stability (RSD,%,n = 3)
Loganic acid	0.25	1.22	0.38
Swertiamarin	0.89	1.63	0.88
Gentiopicroside	0.31	1.58	0.45
Sweorside	0.68	1.88	0.82
Isoorientin	0.55	1.45	0.47

### 3.4 Quantitation of the five components of *G. straminea* from 15 different regions

**Fig. 2:**
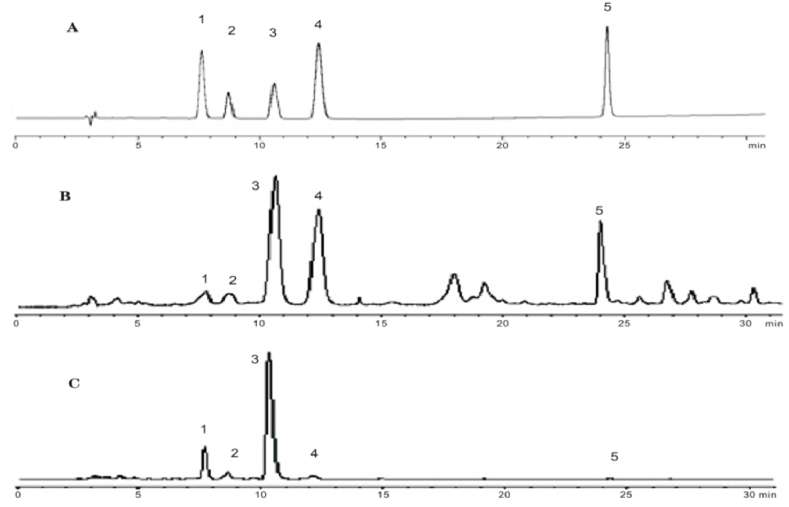
HPLC chromatograms of reference and samples. (A) Mixed standards; (B) Aerial parts of *G. straminea*; (C) Underground parts of *G. straminea*. 1, Loganic acid; 2, Swertiamarin; 3, Gentiopicroside; 4, Sweorside; 5, Isoorientin.

Natural and cultured *G. straminea* were analyzed using the calibration curve of each investigated compound (Table 5). This analysis identified different distributions of the five active components when comparing the aerial parts (Fig. 2B), underground parts (Fig. 2C) and mixed standards (Fig. 2A).

**Table 5:** Content (%) of the five components of *G. straminea* from 15 different regions.

Samples		Loganic acid	Swertiamarin	Gentiopicroside	Sweorside	Isoorientin	Sum	Tatol
MY	Aerial parts	0.375^a^	0.509	3.063	0.044	0.089	4.080	9.182
	Underground parts	___^b^	0.715	4.221	0.073	0.093	5.102	
ZK-1	Aerial parts	0.633	2.053	12.048	0.215	0.347	15.296	22.225
	Underground parts	___^b^	0.961	5.754	0.080	0.134	6.929	
HZ	Aerial parts	0.550	1.501	8.600	0.150	0.270	11.071	21.376
	Underground parts	0.389	1.373	8.260	0.130	0.153	10.305	
NQ	Aerial parts	3.563	1.689	8.580	0.144	0.603	14.579	25.185
	Underground parts	2.941	1.222	6.155	0.085	0.203	10.606	
ZK-2	Aerial parts	1.956	1.320	5.135	0.244	0.462	9.117	15.794
	Underground parts	2.361	0.882	3.255	0.040	0.139	6.677	
TD	Aerial parts	3.523	2.248	8.002	0.122	0.527	14.422	22.074
	Underground parts	1.823	1.113	4.524	0.082	0.110	7.652	
HN-1	Aerial parts	3.897	3.023	6.370	0.062	0.766	14.118	25.525
	Underground parts	4.691	1.706	4.823	0.053	0.134	11.407	
YS-1	Aerial parts	2.371	2.463	4.795	0.077	0.592	10.298	29.406
	Underground parts	5.863	1.751	11.350	0.029	0.115	19.108	
HN-2	Aerial parts	2.332	3.121	7.599	0.083	0.903	14.038	21.715
	Underground parts	1.229	1.462	4.775	0.071	0.140	7.677	
QL-1	Aerial parts	2.661	3.616	8.592	0.071	0.504	15.444	24.516
	Underground parts	2.354	1.566	4.939	0.042	0.171	9.072	
TR-1	Aerial parts	4.409	3.769	8.781	0.075	0.952	17.626	30.541
	Underground parts	3.992	1.756	6.982	0.040	0.146	12.916	
HNP	Aerial parts	4.299	2.959	14.409	0.037	0.550	22.254	51.174
	Underground parts	10.196	2.750	15.783	0.045	0.147	28.921	
QL-2	Aerial parts	4.371	2.245	8.413	0.169	0.366	15.564	31.398
	Underground parts	5.154	1.515	9.030	0.042	0.093	15.834	
TR-2	Aerial parts	3.734	3.328	7.707	0.059	0.807	15.635	24.716
	Underground parts	2.616	1.543	4.585	0.058	0.279	9.0805	
YS-2	Aerial parts	3.378	3.352	9.392	0.136	0.671	16.929	32.942
	Underground parts	4.285	1.944	9.640	0.028	0.116	16.013	

a The date was present as average of three replicates (RSD<2 %).

b Undetected.

### 3.5 Comparison of aerial parts and underground parts of *G. straminea*

**Fig. 3:**
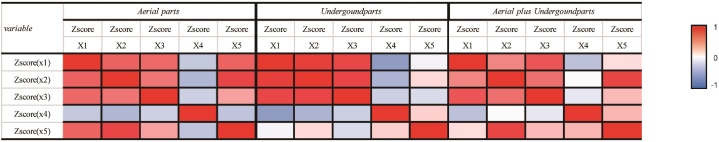
Correlation matrix.

**Fig. 4:**
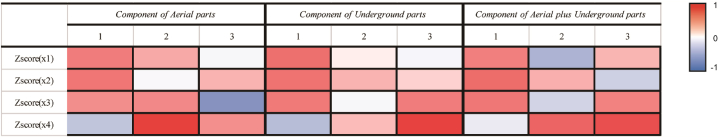
Component matrix.

Procedures of principal component analysis involve the following: 1) Standardization of raw data; 2) Calculation of covariance and correlation coefficient matrix (Fig. 3); 3) Calculation of the eigenvalues and eigenvectors of the covariance matrix (Table 6); and 4) Determination of the principal components combined with professional knowledge to explain the information contained and given by the principal component analysis (Fig. 4, Table 7, Table 8, Table 9) (see Table 10).

**Table 6:** Variance explained and extraction sums of squared loadings.

	Component of Aerial parts			Component of Underground parts			Component of Aerial parts plus Underground parts		
	1 2 3			1 2 3			1 2 3		
Eigenvalues	2.971	0.821	0.712	2.944	1.062	0.781	2.454	1.471	0.722
% of Variance	59.430	16.424	14.237	58.872	21.242	15.630	49.083	29.417	14.445
Cumulative %	59.430	75.854	90.090	58.872	80.114	95.743	49.083	78.500	92.945

Extraction Method: Principal Component Analysis.

**Table 7:** Component equation.

Samples	Component	Component equation
Aerial parts	*F*_*1*_	*F*_*1*_ = *0.487x*_*1*_*+0.524 x*_*2*_*+0.403 x*_*3*_*+0.309x*_*4*_*+0.481 x*_*5*_
	*F*_*2*_	*F*_*2*_ = *0.281x*_*1*_*-0.032 x*_*2*_*+0.430 x*_*3*_*+0.854x*_*4*_*-0.064 x*_*5*_
	*F*_*3*_	*F*_*3*_ = -*0.038x*_*1*_*+0.236 x*_*2*_*-0.660 x*_*3*_*+0.398x*_*4*_*+0.590 x*_*5*_
Underground parts	*F*_*1*_	*F*_*1*_ = *0562x*_*1*_*+0.539 x*_*2*_*+0.507 x*_*3*_*-0.364x*_*4*_*-0.061 x*_*5*_
	*F*_*2*_	*F*_*2*_ = *0.045x*_*1*_*+0.238 x*_*2*_*-0.038 x*_*3*_*+0.210x*_*4*_*+0.947 x*_*5*_
	*F*_*3*_	*F*_*3*_ = -*0.043x*_*1*_*+0.131x*_*2*_*+0.488 x*_*3*_*+0.840x*_*4*_*-0.197 x*_*5*_
Aerial plus Underground parts	*F*_*1*_	*F*_*1*_ = *0.475x*_*1*_*+0.574 x*_*2*_*+0.519 x*_*3*_*-0.093x*_*4*_*+0.408 x*_*5*_
	*F*_*2*_	*F*_*2 = -*_*0.427x*_*1*_*+0.256 x*_*2*_*-0.222 x*_*3*_*+0.623x*_*4*_*+0.561x*_*5*_
	*F*_*3*_	*F*_*3*_ = *0.232x*_*1*_*-0.244 x*_*2*_*+0.465x*_*3*_*+0.740x*_*4*_*-0.352 x*_*5*_

**Table 8:** Component score and composite score ranking of Aerial parts and Underground parts.

NO.	Code	Aerial parts					Underground parts				
		F_1_	F_2_	F_3_	F^a^	ranking	F_1_	F_2_	F_3_	F^b^	ranking
1	MY	−3.338	−2.061	−0.753	−2.428	15	−2.021	−1.338	0.078	−1.462	15
2	ZK-1	−2.224	0.718	0.551	−1.125	13	−1.671	−0.354	0.408	−0.996	14
3	HZ	−1.979	0.301	−0.8715	−1.250	14	−1.458	0.591	2.334	−0.369	10
4	NQ	−0.109	0.734	−0.114	0.039	10	−0.866	1.251	0.351	−0.190	9
5	ZK-2	−2.223	1.218	1.044	−0.972	12	−1.109	−0.531	−1.267	−0.963	13
6	TD	0.050	0.321	−0.150	0.061	9	−1.303	−0.707	0.403	−0.855	12
7	HN-1	1.122	−0.788	0.685	0.634	5	0.343	−0.116	−0.439	0.109	6
8	YS-1	−0.085	−0.755	0.137	−0.154	11	1.966	−0.718	−0.158	0.981	2
9	HN-2	0.997	−0.647	0.897	0.614	6	−0.905	−0.031	0.075	−0.528	11
10	QL-1	0.803	−0.480	−0.320	0.353	7	−0.185	0.441	−0.916	−0.158	8
11	TR-1	2.296	−0.235	0.766	1.434	1	0.733	0.014	−0.552	0.349	5
12	HNP	2.227	0.415	−2.189	1.080	2	4.372	0.554	1.036	2.854	1
13	QL-2	−0.144	1.256	−0.382	0.066	8	1.067	−1.162	−0.052	0.374	4
14	TR-2	1.550	−0.651	0.491	0.884	3	−0.561	2.737	−0.937	0.103	7
15	YS-2	1.058	0.655	0.207	0.765	4	1.598	−0.631	−0.363	0.751	3

a *F* = 0.594 *F*_*1*_ + *0.164 F*_*2*_*+0.142 F*_*3*_.

b *F* = 0.589 *F*_*1*_ + *0.212 F*_*2*_*+0.156F*_*3*_.

**Table 9:** Component score and composite score ranking of aerial parts plus underground parts.

NO.		Code	F_1_	F_2_	F_3_	F^a^	ranking
1	Aerial parts	MY	−2.623	−0.589	−0.803	−1.577	30
2		ZK-1	−0.951	2.060	1.264	0.322	13
3		HZ	−0.878	0.825	1.027	−0.040	15
4		NQ	0.435	0.889	0.791	0.589	10
5		ZK-2	−1.157	2.221	1.708	0.332	12
6		TD	0.604	0.685	0.337	0.547	11
7		HN-1	1.358	0.768	−1.208	0.718	8
8		YS-1	0.441	0.698	−0.728	0.317	14
9		HN-2	1.454	1.546	−1.107	1.009	5
10		QL-1	1.438	0.580	−0.691	0.777	7
11		TR-1	2.554	1.302	−1.078	1.481	1
12		HNP	2.544	−0.674	0.069	1.061	4
13		QL-2	0.543	0.689	1.351	0.664	9
14		TR-2	1.819	0.838	−1.192	0.967	6
15		YS-2	1.715	1.388	0.251	1.287	2
16	Underground parts	MY	−2.415	−0.203	−0.328	−1.293	29
17		ZK-1	−1.937	−0.084	−0.111	−0.991	27
18		HZ	−1.199	0.376	0.871	−0.352	18
19		NQ	−0.937	−0.438	0.185	−0.562	21
20		ZK-2	−1.819	−0.844	−0.773	−1.253	28
21		TD	−1.684	−0.345	−0.087	−0.941	26
22		HN-1	−0.517	−1.064	−0.308	−0.612	22
23		YS-1	0.943	−2.097	0.542	−0.076	16
24		HN-2	−1.487	−0.208	−0.396	−0.848	25
25		QL-1	−1.041	−0.686	−0.707	−0.815	24
26		TR-1	−0.222	−1.195	−0.249	−0.496	20
27		HNP	3.368	−2.780	1.627	1.070	3
28		QL-2	0.166	−1.742	0.365	−0.378	19
29		TR-2	−0.924	−0.314	−0.651	−0.640	23
30		YS-2	0.409	−1.602	0.030	−0.266	17

a *F* = 0.491 *F*_*1*_ + *0.294F*_*2*_*+0.144 F*_*3*_.

**Table 10:** Matrix correlation of altitude and analyzed constituents.

		1	2	3	4	5
Altitude	r	0.414	0.188	−0.037	0.082	0.295
	p	0.125	0.502	0.897	0.771	0.286

1 Loganic acid, 2 Swertiamarin, 3 Gentiopicroside, 4 Sweroside, 5 Isoorientin.

## 4 Discussion

In the Pharmacopoeia, *G. straminea* is regarded as one of the "Ma Hua Jiao", and the minimum content of gentianoside in its dried roots must be 2.0 % [39]. According to the results of our study, the minimum content of gentianosides in *G. straminea* populations was not less than 2.0 % (Table 5). This finding supports the use of all these populations as an original medicinal resource. This analysis of the constituents of *G. straminea* also suggested a plausible explanation as to why Tibetan doctors utilize the whole plant while Chinese doctors typically only the root of *G. straminea*. The principal component analysis was performed on data from the aerial and underground parts of *G. straminea*, respectively. After three components were extracted, the cumulative variance contributions from the two parts exceeded 85 % and satisfied the minimum requirements of analysis (Table 6). This indicated that the extracted components were sufficiently representative of the total information related to the five pharmacological components. Combining the results calculated by principal component analysis of the aerial and underground parts of *G. straminea*, respectively (Table 7, Table 8), confirmed that the concentrations of the five active components did not differ significantly between the aerial and underground parts from different regions. In brief, the principal component analysis is a single composite index representing all of the determined components from *G. straminea*; it is a collection of components and playing a leading role.

Unfortunately, calculations of data comparing the main components of the aerial and underground parts were insufficient to explain how the aerial parts of *G. straminea* were able to entirely replace the underground parts. Therefore, another principal component analysis was performed to comprise and unify all measured data from both the aerial parts and underground parts under a standard (Table 9). The results indicated that component *F*_*1*_ represented loganic acid, swertiamarin and gentiopicroside; component *F*_*2*_ represented sweorside; and component *F*_*3*_ represented isoorientin. Because of the pharmacological activity associated with loganic acid, swertiamarin and gentiopicroside, component *F*_*1*_ represented the major constituents responsible for *G. straminea* activity related to analgesic, anti-inflammatory, and febrifuge properties, as well as its protection against hepatitis. Sweorside can function as an anticonvulsant, so component *F*_*2*_ represented pluralities of constituents possessing the properties of an anticonvulsant in *G. straminea*. The pharmacological activity of isoorientin includes anticoagulation, anti-bacterial and anti-oxidation, so component *F*_*3*_ represented several compounds exhibiting the same pharmacological activity in *G. straminea*. Analysis of these three combined principal components and calculation of composite scores indicated higher ranking of the aerial parts from different regions, compared to the underground parts, thereby indicating that the aerial parts can replace the underground parts.

### 4.1 Comparison of different regions of *G. straminea*

Hierarchical clustering analysis was performed based on the data determined by HPLC of aerial parts of *G. straminea* from 15 different regions. Between-groups Linkage, a very efficient method for the analysis of variance between clusters, was applied, and Squared Euclidean distance was selected as a measurement. Principal component stacked contrast analysis and correlation analysis of the concentrations of the five components in relation to elevation were also carried out.

**Figs. 5 and 6:**
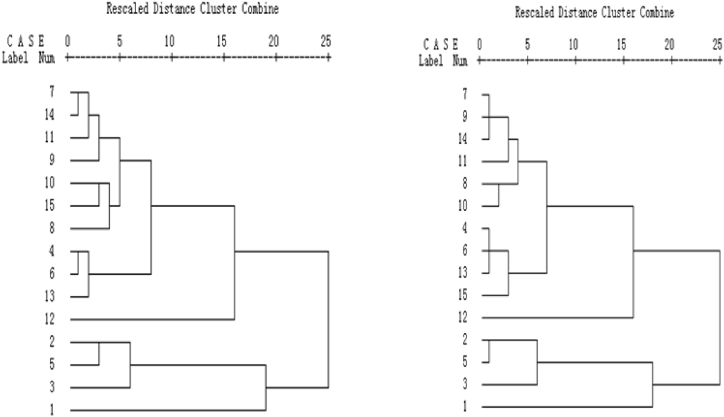
Dendrograms of hierarchical cluster analysis for the 15 tested samples of aerial parts of *G. straminea*. The hierarchical clustering was done using SPSS software. A method called average linkage between groups was applied, and Squared Euclidean distance was selected as the measurement. In figure 5 (left), the dendrogram represents five active components, derived from data determined by HPLC of the tested 15 *G. straminea* samples. In figure 6 (right), the dendrogram represents data from a score of three components extracted from aerial parts of the tested 15 *G. straminea*.

**Fig. 6:**
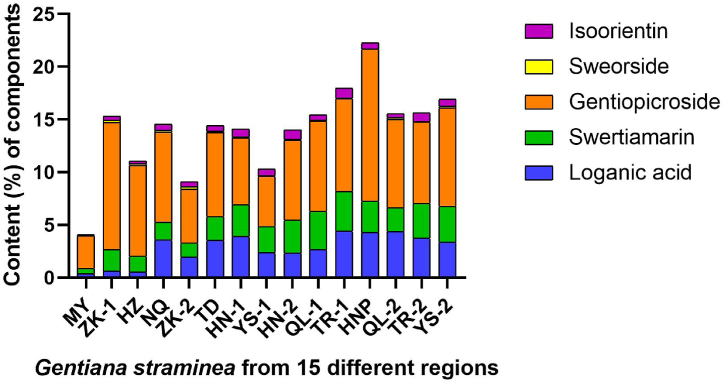
Stacked histogram of five major pharmacological constituents of the above ground portion of *G. straminea*.

The dendrogram of clustering analysis showed that the *G. straminea* populations could be divided into four main clusters quality production regions according to the content of four marker compounds during the harvest season(Fig. 5): HN-1(7), TR-2(14), TR-1(11), HN-2(9), QL-1(10), YS-2(15), YS-1(8), NQ(4), TD(6), and QL-2(13) are cluster one; ZK-1(2), ZK-2(5), and HZ(3) are cluster two; MY(1) is cluster three; and HNP(12) is cluster four. Generally, the results of cluster analysis are more closely realistic. Interestingly, of the 15 populations, four populations contained superior constituent content to the others and these populations (Fig. 5). This suggests that there are several “geo-authentic” production regions of “Ma Hua Jiao”. The regions of HN-1(7), TR-2(14), TR-1(11), and HN-2(9) in cluster one is indeed good growth areas for *G. straminea*, and the determined contents of each component in *G. straminea* from those regions are higher. A sub-group consisting of NQ (4), TD (6), QL-2 (13), QL-1 (10), YS-2 (15), and YS-1 (8) from cluster one more closely approached the results suggested by the composite score of principal component analysis from aerial parts of *G. straminea*. HNP (12) represents a cluster for cultivation. In addition, using data from the component score of three components extracted from aerial parts, results of the hierarchical cluster analysis of the tested 15 samples were similar to those derived from the five active components (Fig. 6). Furthermore, stacked analyses of the five main components of *G. straminea* from the 15 regions were performed (Fig. 6), and it is clear and intuitive that HNP (12) corresponds to cluster four in Fig. 5, which is a well-grown region although it is an artificially cultivated sample, and that MY (1) corresponds to cluster three in Fig. 5, which is the region with the least amount of *G. straminea* components. It was also clear to see that gentianoside was the highest (see Fig. 6).

**Fig. 7:**
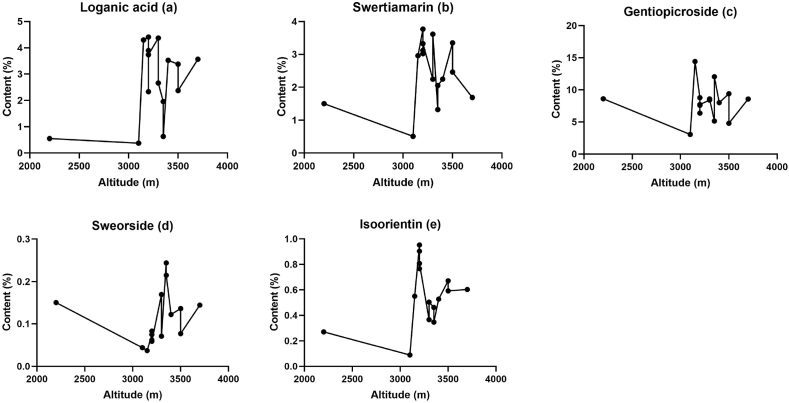
Variations in the mean content of the five active constituents (a) Loganic acid, (b) Swertiamarin, (c) Gentiopicroside, (d) Swerosideand and (e) Isoorientin, over the altitude gradient.

*G. straminea* was characterised by a combination of dichogamy and herkogamy [40] and had high population genetic diversity [41]. And correlation analyses were carried out to determine the relationship between the concentrations of the five components and altitude. Although the concentrations of the loganic acid (Fig. 7a), swertiamarin (Fig. 7b), gentiopicroside (Fig. 7c), sweorside (Fig. 7d), and isoorientin (Fig. 7e) fluctuated between altitudes, Pearson correlation showed no significant relationship between the concentrations of the five components and altitude. The correlation coefficients were 0.414, 0.188, −0.037, 0.082 and 0.295, respectively (Table 9). Our results suggest that the populations of this species also have significant chemical variation among different populations on the Qinghai-Tibetan Plateau. Chinese medicinal material cultured in different localities is believed to differ in therapeutic potency [42]. Although genetic differences have been suggested to guide research and conservation of *G. straminea* [43], genetic proof is very limited and therefore concentrations of bioactive components should also be considered. Due to the high vulnerability of the region to climate change and human activities, over-harvesting of plants may lead to population scarcity. It is important to pay attention to the concentration of bioactive components when implementing policies for harvesting and conserving wild species, especially in areas where they are more suitable for growth. Therefore, the findings of this paper provide a strong reference value for sustainable species development and conservation.

## 5 Conclusions

This work first developed a simple and reliable HPLC method for simultaneous, quantitative determination of five active compounds in the aerial parts of *G. straminea*. Active constituents’ analysis of *G. straminea* showed that the aerial parts are slightly better than the underground parts, and the former can replace the latter in traditional medicines. Gentianolide was the most abundant constituent, with all populations having levels well above national standard levels. Finally, Hierarchical clustering analysis suggests that suitable growth areas for the above-ground parts of broad-leaved gentian were identified based on the content of labelled compounds. Although there was no significant relationship between the concentrations of the five components and altitude, our findings provide definitive phytochemical evidence for the conservation of resources and more careful utilization of this traditional Tibetan medicinal herb. Based on the analyses of the five constituents, it also seems reasonable to establish quality standards within the Tibetan Plateau Taoist medicinal culture region.

## 6 Limitations

This study has potential limitations. It should be noted that this paper only represents investigation of five active compounds from *G. straminea*, and it is slightly one-sided on behalf of all the ingredients. However, this study represents a preliminary attempt to apply statistical approaches towards solving practical issues, and further study will be needed in the future.

## CRediT authorship contribution statement

**Junlei Hao:** Writing – review & editing, Methodology, Investigation, Data curation. **Jiang Zhou:** Data curation. **Pengcheng Lin:** Writing – review & editing. **Jiang Wu:** Methodology, Conceptualization.

## Declaration of competing interest

The authors declare that they have no known competing financial interests or personal relationships that could have appeared to influence the work reported in this paper.
